# Supplementary material for: Right On Time: Ultrafast Charge Separation Before Hybrid Exciton Formation
Source: Adv Sci (Weinh). 2024 Jun 14;11(31):2403765. doi: 10.1002/advs.202403765 (PMC11336905; doi:10.1002/advs.202403765)
Supplement: Supplementary file 1 — Supporting Information [file ADVS-11-2403765-s001.pdf]

ADVANCED SCIENCE
Open Access

Supporting Information

for *Adv. Sci.*, DOI 10.1002/advs.202403765

Right On Time: Ultrafast Charge Separation Before Hybrid Exciton Formation

Lukas Gierster, Olga Turkina*, Jan-Christoph Deinert, Sesha Vempati, Elsie Baeta, Yves Garmshausen, Stefan Hecht, Claudia Draxl and Julia Stähler*

SUPPORTING MATERIAL Right on time: Ultrafast Charge Separation Before Hybrid Exciton Formation

Lukas Gierster, Olga Turkina*, Jan-Christoph Deinert, Sesha Vempati, Elsie Bowen Dodoo, Yves Garmshausen, Stefan Hecht, Claudia Draxl, Julia Stähler*

L. Gierster, Y. Garmshausen, S. Hecht, J. Stähler
Department of Chemistry, Humboldt-Universität zu Berlin, Brook-Taylor-Str. 2, 12489 Berlin, Germany
L. Gierster, J.-C. Deinert, S. Vempati, E. Bowen Dodoo, J. Stähler
Department of Physical Chemistry, Fritz-Haber-Institut der Max-Planck-Gesellschaft, Faradayweg 4-6, 14195 Berlin, Germany
O. Turkina, C. Draxl
Department of Physics, Humboldt-Universität zu Berlin, Newtonstr. 15, 12489 Berlin, Germany
S. Hecht, C. Draxl
Center for the Science of Materials Berlin, Humboldt-Universität zu Berlin, Zum Großen Windkanal 2, 12489 Berlin, Germany
*Email Addresses: lukas.gierster@hu-berlin.de, turkina@physik.hu-berlin.de

1 Delay-dependent photostationary states - modeling

The main text reports on the observation of a photo*stationary* state that exhibits a dependence on the pump-probe time delay, which may appear counter-intuitive. In the following, we present a simple model description of the proposed mechanism behind it, demonstrating that photostationary states can vary for different time delays and that the time delay dependence reflects the population dynamics on ultrafast timescales.

The model is based on the population dynamics of three states, the ground state (GS), the photostationary state (HX), and the so-called intermediate state (IS). The latter is a simplification that subsumes all states that are populated by the electron on its journey to the HX. As these dynamics occur on femtosecond timescales, they are irrelevant for the slow dynamics of the photostationary state. As illustrated by Fig. S1a, the pump pulse hν_1 populates IS with a probability α, which decays to HX with a time constant $\kappa = 100$ ps. As in the experiment, the probe laser pulse leads to photoemission from HX and IS, reducing the populations by β and γ, respectively. As observed in the experiment, we include a lifetime τ of HX that exceeds the inverse repetition rate of the laser system.

Before going into the details of the model, we illustrate the origin of the delay dependence of the photostationary state by comparison of the population dynamics of HX (green) and IS (grey) for two different time delays in Fig. S1b and c. In b, the probe laser pulse (red) arrives at a late time delay Δt_1 when all the IS population has already transferred to HX. The HX population is reduced (with the probability β) by $\delta(\Delta t_1)$ and the remainder contributes to the photostationary state population n_{HX}^{∞} that is probed by subsequent laser pulse pairs. On the contrary, in c, the probe pulse arrives *before* all electrons have transferred to HX. In this scenario, hν_2 depopulates both, HX *and* IS with β and γ, respectively. This leads to a different reduction of the population $\delta(\Delta t_2)$ than in the previous case, resulting in a different contribution to the photostationary state intensity n_{HX}^{∞}.

We can detail these thoughts using mathematical relationships; we define α, β and γ as excitation probabilities between 0 and 1. The population increase induced by the pump laser pulse hitting the sample at the time T_0 is

$$n_{IS}(T_0) = \alpha\ n_{GS}. \tag{1}$$

The population of the IS then transfers to HX. The temporal evolution on ultrafast timescales (κ=100 ps) is given by

$$n_{IS}(t) = \alpha\ n_{GS}\ e^{-\Delta t/\kappa} \tag{2}$$

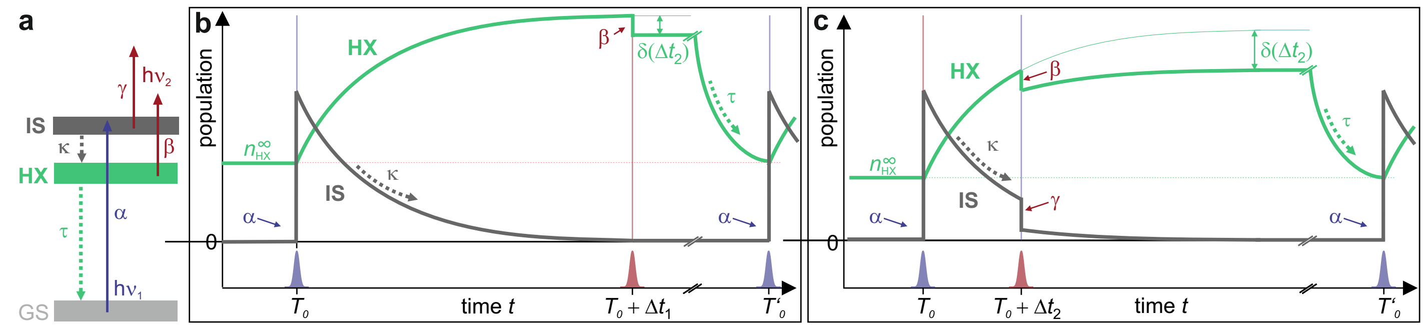

Figure S1: Illustration of the time-dependence of the photostationary state. a) Relevant states and transitions. b,c) Population dynamics of HX and IS for two different positive pump-probe delays Δt, see text for details.

$$n_{HX}(t) = \alpha\ n_{GS}\ (1 - e^{-\Delta t/\kappa}) + n^0_{HX} \tag{3}$$

Here, n^0_{HX} is the population of the HX remaining from preceeding excitation events due to the long lifetime of this state. The probe laser pulse arrives at $t = T_0 + \Delta t$, where T_0 marks the arrival of the pump pulse. In the following we are interested in the calculation of the photostationary equilibrium, which is determined by the temporal evolution on macroscopic timescales (on the order of or larger than the inverse laser repetition rate). This is determined by the boundary value of the HX population after the probe pulse hit the sample and after all population has transferred from IS to HX (cf. Fig. S1b,c).

$$n^k_{HX} = (1 - \beta)\ n^0_{HX}(\Delta t) + (1 - \gamma)\ n_{IS}(\Delta t), \tag{4}$$

Here, we introduce the index k, which denotes the counter of the pump-probe pulse pair. Note that the equation contains n^0_{HX}, the population of the HX remaining from preceeding excitation events, which is necessary to account for the long lifetime of HX. We assume that the HX population decays exponentially in between two pump-probe pairs with τ on the order of the inverse laser repetition rate $1/R$. As the time between subsequent pulse pair equals $\frac{1}{R}$, we can write:

$$n^0_{HX} = n^{k-1}_{HX}\ e^{-1/R\tau} \tag{5}$$

With this, equation 4 becomes a recursive equation.

The last step is the definition of a photostationary state, which means that the population remains constant between suceeding pump-probe pulse pairs:

$$n^\infty_{HX} := n^{k-1}_{HX} = n^k_{HX} \tag{6}$$

From this, we can deduce the delay-dependent photostationary population

$$n^\infty_{HX} = \alpha\ n_{GS} \frac{(\gamma - \beta)(1 - e^{-\delta T/\kappa}) + (1 - \gamma)}{1 - (1 - \beta)e^{-1/R\tau}} \tag{7}$$

This equation describes an exponential rise with the time constant κ if $\gamma > \beta$ and shows that the ultrafast population dynamics of a state with a lifetime on the order of the inverse repetition rate of the laser system can be reflected in the photostationary intensity of this state. We conclude that there is no conflict between the observation of photoinduced dynamics on ultrafast timescales and the conclusion that these relate to a photostationary state.

Note that this is a simplified version of the model to illustrate the general principle. For simplification, we have neglected that the probe laser beam also creates HX population and that the pump laser beam also depopulates HX by photoemission. To account for these effects, (de)population parameters need to be included in the above derivation. This complicates the mathematics, but the resulting delay-dependency remains identical as discussed in detail in Ref. [1].

2 Theoretical methodology and computational details

We model the interface nP-Py/ZnO by adopting four layers of ZnO fully covered by an upright-standing monolayer of pyridine molecules for $n = 0$ that is extended by one ($n = 1$) or two ($n = 2$) phenyl rings along the molecular plane of pyridine. A detailed description of this setup can be found in Ref. [2].

Density-functional theory (DFT) constitutes the starting point of our calculations with the local-density approximation (Perdew-Wang parametrization [3]) serving as the exchange-correlation functional. Having obtained the ground-state properties, we make use of the Kohn-Sham (KS) eigenvalues and orbitals as input for our investigations of the excited-state properties within the framework of many-body perturbation theory. The quasi-particle (QP) band structure is obtained applying the G_0W_0 approximation [4, 5], and the Bethe-Salpeter equation (BSE) [6, 7] is solved in order to capture excitonic effects in the optical spectra and investigate arising excitations individually. Formulated as an eigenvalue problem the BSE reads

$$\sum_{v'c'\mathbf{k}'} H_{vc\mathbf{k},v'c'\mathbf{k}'}\, A^{\lambda}_{v'c'\mathbf{k}'} = E^{\lambda} A^{\lambda}_{vc\mathbf{k}}. \tag{8}$$

The effective Hamiltonian consists of terms accounting for vertical transitions from the valence (v) to the conduction (c) band region, the electron-hole exchange and the screened Coulomb interaction, the latter giving rise to the formation of bound excitons. The eigenvalues E^{λ} are the excitation energies and the eigenvectors $A^{\lambda}_{vc\mathbf{k}}$ can be interpreted as coupling coefficients, carrying information about the mixing of transitions. We visualize individual excitons in real space using the exciton wave function and in the reciprocal space in terms of excitonic weights. The excitonic wave function can be expressed as a linear combination of the KS wave functions weighted by the coupling coefficients $A^{\lambda}_{vc\mathbf{k}}$

$$\Phi^{\lambda}(\mathbf{r}_e, \mathbf{r}_h) = \sum_{vc\mathbf{k}} A^{\lambda}_{vc\mathbf{k}} \psi^{*}_{v\mathbf{k}}(\mathbf{r}_h)\psi_{c\mathbf{k}}(\mathbf{r}_e) \tag{9}$$

while the excitonic weights $w^{\lambda}_{ck} = \sum_v |A^{\lambda}_{vc\mathbf{k}}|^2$ and $w^{\lambda}_{vk} = \sum_c |A^{\lambda}_{vc\mathbf{k}}|^2$ express the composition of the exciton in terms of contributing QP bands.

All calculations are performed using the all-electron full-potential package **exciting** [8], implementing linearized augmented planewave plus local-orbitals methods. The muffin-tin radii used for the involved atomic species are $R^{\mathrm{H}}_{\mathrm{MT}} = 0.8$ bohr, $R^{\mathrm{N}}_{\mathrm{MT}} = R^{\mathrm{C}}_{\mathrm{MT}} = 1.2$ bohr, $R^{\mathrm{Zn}}_{\mathrm{MT}} = R^{\mathrm{O}}_{\mathrm{MT}} = 1.6$ bohr. For the ground-state calculations, the sampling of the Brillouin zone (BZ) is performed on a $10 \times 10 \times 1$ **k**-grid ($n = 0$, 1) and $12 \times 12 \times 1$ **k**-grid ($n = 2$). A basis-set cutoff $|\mathbf{G} + \mathbf{k}|_{max}$ of 5 ($n = 0$) and 4.375 ($n = 1, 2$) is used. To compensate for the artificial electric field across the slab caused by the asymmetric adsorption geometry a dipole correction is applied. The calculations of QP corrections to the KS eigenvalues within the G_0W_0 approximation [9] include 1000 empty bands, and the BZ is sampled on a $4 \times 4 \times 1$ **k**-grid. Wannier interpolation is used to visualize the band structure. The BSE [10, 11] is solved within the Tamm-Dancoff approximation. 150 conduction bands are included in the calculation of the response function and the screened Coulomb potential. 41 (54, 45) occupied and 25 (20, 20) unoccupied bands for $n = 0$ ($n = 1$, 2) on a $12 \times 12 \times 1$ shifted **k**-grid are taken into account in the construction of the BSE Hamiltonian. VESTA software [12] is used to visualize atomic structures and isosurfaces.

Integration of the electron density distribution of the HX presented in the main text allows us to determine its extension. Figure S2 displays the charge density along out-of-plane and in-plane directions. Panel **a** reveals that the electron resides on ZnO as we observe four (double) peaks corresponding to four ZnO layers, with the highest contribution arising from the region close to the interface. The three topmost ZnO layers encompass 78 % of the electron density within 1 nm. Laterally, the extension within 1 nm reaches 60 % of the electron density.

Figure S3 shows excitons in 1P-Py/ZnO and 2P-Py/ZnO systems that are of similar character to the ones presented in the main text (cf. Fig. 2) as they manifest transitions from molecular to ZnO-dominated bands around the Γ point. As we display the excitonic weights on the same footing, we note that the Γ-point contributions to these excitons are much smaller compared to the hybrid exciton displayed in the main text. This reflects the fact that the latter is strongly bound and localized, with the contributions stemming

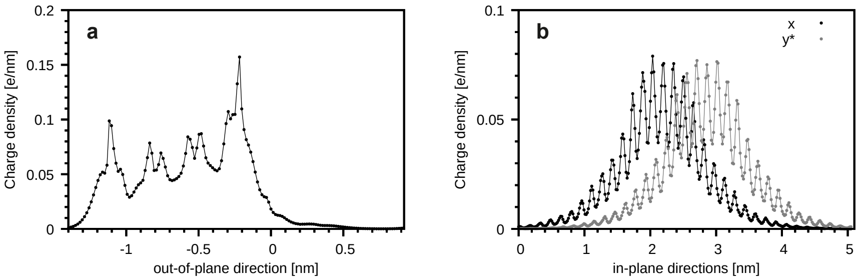

Figure S2: **Integrated electron density** of the HX (cf. Fig. 2 d,e) in **a,** out-of-plane (nitrogen atom at 0 nm) and **b,** in-plane directions.

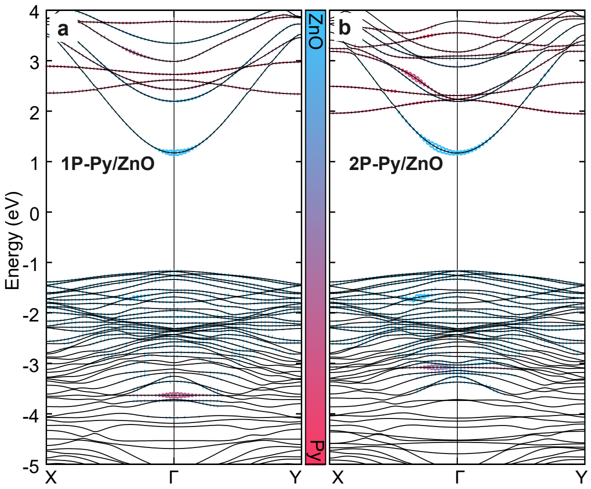

Figure S3: **Electronic structure and excitonic weights** in **a,** 1P-Py/ZnO and **b,** 2P-Py/ZnO as calculated by DFT+*GW* and hybrid exciton contributions based on BSE.

almost exclusively from the Γ-point. By contrast, in Figure S3, transitions from various regions of the BZ contribute slightly to the formation of the excitons, leaving the Γ-point transition not as prominent. An in-depth discussion of the characteristics of excitations across nP-Py/ZnO interfaces will be conducted in a subsequent paper. There it will also be shown that the main characteristics of the electronic structure do not change when going from four to six ZnO layers. From this, we don't expect major changes in the exciton binding energies when considering thicker slabs.

3 Experimental setup

The experimental setup is sketched in Figure S4.

References

[1] L. Gierster. Zno: Ultrafast photodoping - ultrashort and metastable photoinduced metallization of the zno(10-10) surface, **2021**.

[2] O. Turkina, D. Nabok, A. Gulans, C. Cocchi, C. Draxl. *Advanced Theory and Simulations* **2019**, *2*, 2 1800108.

[3] J. P. Perdew, Y. Wang. *Phys. Rev. B* **1992**, *45* 13244.

[4] L. Hedin. *Phys. Rev.* **1965**, *139* A796.

[5] M. S. Hybertsen, S. G. Louie. *Phys. Rev. B* **1986**, *34* 5390.

[6] E. E. Salpeter, H. A. Bethe. *Phys. Rev.* **1951**, *84* 1232.

[7] G. Strinati. *La Rivista del Nuovo Cimento* **1988**, *11* 1.

[8] A. Gulans, S. Kontur, C. Meisenbichler, D. Nabok, P. Pavone, S. Rigamonti, S. Sagmeister, U. Werner, C. Draxl. *Journal of Physics: Condensed Matter* **2014**, *26*, 36 363202.

[9] D. Nabok, A. Gulans, C. Draxl. *Phys. Rev. B* **2016**, *94* 035118.

[10] S. Sagmeister, C. Ambrosch-Draxl. *Phys. Chem. Chem. Phys.* **2009**, *11* 4451.

[11] C. Vorwerk, B. Aurich, C. Cocchi, C. Draxl. *Electronic Structure* **2019**, *1* 037001.

[12] K. Momma, F. Izumi. *Journal of Applied Crystallography* **2011**, *44*, 6 1272.

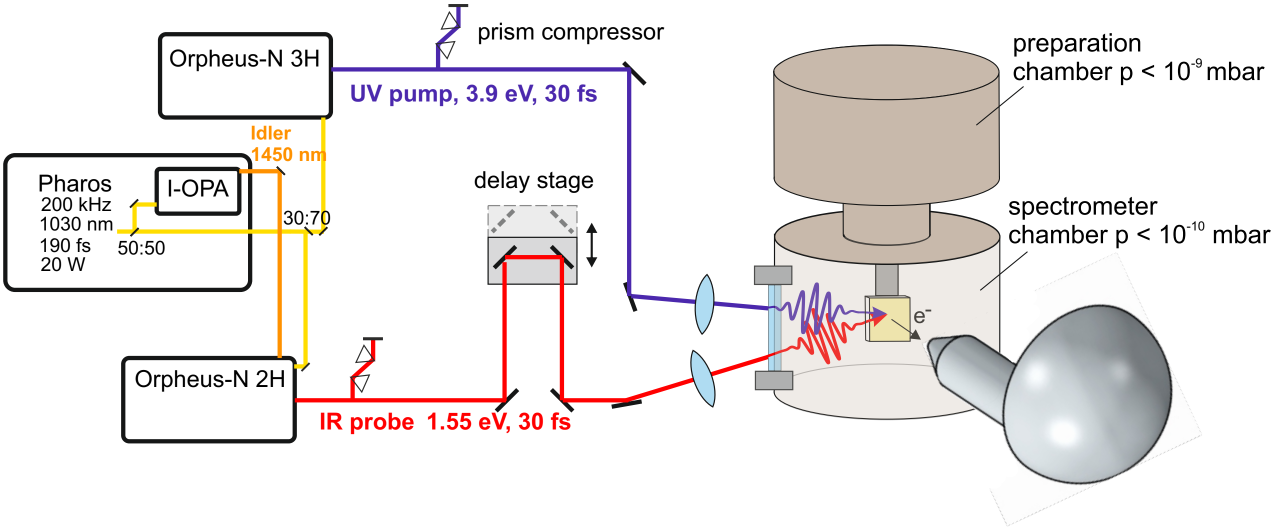

Figure S4: Experimental setup to conduct time-resolved 2PPE measurements. The laser system by Lightconversion is based on the Pharos regenerative amplifier (20 W, 200 kHz repetition rate, 1030 nm center wavelength), which feeds two non-linear optical parametric amplifiers (Orpheus-N 2H, Orpheus-N 3H). The Orpheus 2H white light is generated by the idler (1450 nm) of an internal optical parametric amplifier (IOPA) inside the Pharos. The Orpheus 3H delivers the UV pump pulses for intramolecular excitation, and the Orpheus 2H generates the IR probe beam. For interfacial excitation, the Orpheus 2H delivers the pump beam and the Orpheus 3H is tuned to deliver the IR probe laser pulses. The delay between the two beams is controlled via a delay stage. Both beams are focused into the UHV chamber. The photoelectrons are detected by a hemispherical energy analyser. The preparation chamber contains the Knudsen cell, a quartz microbalance, a sputtergun and a LEED (not shown).
